# Sequential Antioxidants Foliar Application Can Alleviate Negative Consequences of Salinity Stress in *Vicia faba* L.

**DOI:** 10.3390/plants10050914

**Published:** 2021-05-02

**Authors:** Wael M. Semida, Taia A. Abd El-Mageed, Reham M. Abdalla, Khaulood A. Hemida, Saad. M. Howladar, Ahmed A. A. Leilah, Mohamed O. A. Rady

**Affiliations:** 1 Horticulture Department, Faculty of Agriculture, Fayoum University, Fayoum 63514, Egypt; 2 Soil and Water Department, Faculty of Agriculture, Fayoum University, Fayoum 63514, Egypt; taa00@fayoum.edu.eg; 3 Vegetable Crops Department, Faculty of Agriculture, Assiut University, Assiut 71526, Egypt; reham.abdalla@aun.edu.eg; 4 Botany Department, Faculty of Science, Fayoum University, Fayoum 63514, Egypt; kah00@fayoum.edu.eg; 5 Department of Biology, College of Science, University of Jeddah, Jeddah 21589, Saudi Arabia; smhowladar@uj.edu.sa; 6 Agronomy Department, Faculty of Agriculture, Mansoura University, Mansoura 35516, Egypt; whdany2003@yahoo.com; 7 Agronomy Department, Faculty of Agriculture, Fayoum University, Fayoum 63514, Egypt; mer00@fayoum.edu.eg

**Keywords:** *Vicia faba*, sequential antioxidants, salinity stress, photosynthetic efficiency, antioxidant activities

## Abstract

Salinity is one of the most limiting abiotic stresses in agricultural productivity. Exogenously applied antioxidants successfully enabled salt-stressed plants to cope with stress. Two-season field experiments were conducted consecutively in 2016/17 and 2017/18 to study the effects of foliar applications of singular (ascorbate, AsA; proline, Pro; and glutathione, GSH) or sequential (AsA-Pro-GSH and GSH-Pro-AsA) antioxidants on growth, yield, physio-biochemical attributes, and enzymatic and non-enzymatic antioxidative defense system of *Vicia faba* L. (CV. Sakha-1) plants grown under saline soil conditions (EC = 4.53 dS m^−1^). Under soil salinity conditions, AsA, Pro, or GSH-Pro-ASA improved growth and productivity, photosynthesis efficiency, stomatal conductance (gs), plant water status, as well as enzymatic and non-enzymatic antioxidants. However, sequential AsA-Pro-GSH foliar application followed by singular GSH significantly exceeded all other treatments (i.e., AsA, Pro, and GSH-Pro-AsA), improving growth characteristics (shoot length, shoot fresh and dry weights, and leaves area), photosynthesis efficiency, stomatal conductance, plant water status, and yield and its components (green pods weight/plant^−1^, green pods yield/hectare^−1^, and seed yield/hectare^−1^), as well as enzymatic (ascorbate peroxidase, catalase, superoxide dismutase, and glutathione reductase) and non-enzymatic (AsA, GSH, Pro, phenolic aglycone, phenolic glycosides) antioxidants compared to control. Overall, our results clearly demonstrate that sequential AsA-Pro-GSH foliar application has a positive effect on salt-stressed *Vicia faba* plants.

## 1. Introduction

With the consequences of global warming and the rapidly increasing world population, there is a growing need for increasing and securing crop yields [1,2]. Soil salinity, however, is one of the most common environmental threats affecting agricultural productivity [1,3,4,5]. Salinity causes growth reductions and yield losses in most of the crops worldwide especially in arid and semi-arid areas [6,7,8,9]. Salinity inhibits plant growth due to both the osmotic stress caused by the existence of salts in the soil solution and by the ionic-specific effects caused by the accumulation of salts in the transpiring leaves [7,10,11,12]. Reactive oxygen species (ROS) generation is a common consequence of salt stress. Salt stress may induce closure of stomata which results in a lowering of the intracellular CO_2_ concentration in leaves, impeding carbon fixation, resulting in excessive excitation energy in the chloroplasts [1,7,13,14].

ROS are highly active by-products that have an essential signaling role in plants [15,16,17]. Low levels of ROS act in signaling the adaptation responses to biotic and abiotic stresses, and for regulating plant growth and development, whereas elevated amounts of ROS are toxic and cause oxidative stress [6,18,19]. A maintained balance between ROS production and scavenging is important for sustained cellular and plant functions [6,15,19,20]. The antioxidant defense system has a pivotal role in controlling the ROS production–scavenging balance and in protecting plants from damage caused by the oxidative stress [15,18,20]. Enzymatic components of the antioxidant system include ascorbate peroxidase (APX), peroxiredoxin (PRX), dehydroascorbate reductase (DHAR), glutathione-S- transferase (GST), glutathione reductase (GR), superoxide dismutase (SOD), catalase (CAT), and guaiacol peroxidase (GPX). The non-enzymatic components include reduced glutathione (GSH), ascorbate (AsA), proline (Pro), and others such as α-tocopherol, flavonoids, phenolics, and carotenoids [15,18,19,20,21].

AsA is a powerful antioxidant and is the most prevalent one in almost all plant tissues, especially in leaves (almost 5−10 times higher than glutathione) [19,22]. It has several functions as an antioxidant and as an enzyme cofactor in plants [22,23]. It functions as a cofactor for the biosynthesis of some plant phytohormones and their transduction pathways [24]. It has a key role in the regulation of cell division, elongation, and consequently growth and development [22,25,26,27]. It may influence the expression of many defense genes and the genes responsible for plant growth [24,28,29,30].

GSH is a main non-enzymatic antioxidant that has a small intracellular thiol group in plant tissues [31]. This thiol group makes GSH suitable for many biochemical processes such as sulfur metabolism and storage, transport and alterations of various hormones, and regulation of enzyme activities [32]. It also moderates processes such as gene expression, synthesis of proteins, cell proliferation, cell growth, development, and senescence [15,21,33]. It reversibly shifts between its reduced (GSH) and oxidized forms (GSSG) because of the cysteine sulfur group in redox reactions. It is a member of the AsA–GSH cycle for the ascorbate generation and consequently has a role in the defense system of plants. GSH protects the proteins from the oxidative denaturation under the stressful environmental conditions [23,26,34,35,36]. Interestingly, GSH functions in chelating toxic metals to be sequestered in the vacuoles [32,37,38].

Osmotic adjustment of the cells is the first adaptive response to water and salt stresses [39,40,41,42]. Accumulation of compatible solutes in the cytosol is one of the tolerance mechanisms to salinity stress [43,44,45,46]. Proline (Pro) is a low-molecular-weight amino acid and is one of the compatible and non-toxic osmolytes that acts in the osmotic regulation between the cytosol and the vacuoles [44,47]. Different plant species have different accumulation levels of proline in plants and it can be 100 times higher in some stressful conditions compared to the control conditions [48]. Pro is an important antioxidant that acts as a free radical scavenger in plants too. Pro accumulation helps in the balance of the cellular redox status, protects proteins and membrane structures, and serves as a molecular chaperone [41,48].

Faba bean (*Vicia faba* L.) is a very nutritious grain legume that provides 20–36% of protein supply in human and animal diet [49]. Legumes can be sustainable as they can fix nitrogen through their nodules, facilitating their growth in poor-nitrogen soils, and can be used in saline-affected lands to help improve soil fertility. However, salinity can affect legume growth, nodulation, rhizobium symbiosis, and nitrogen fixation [50]. Legumes are classified as sensitive (or only moderately tolerant) to salt stress [51]. Faba bean is categorized as moderately sensitive to salinity [52]. In Egypt, no faba bean cultivars were released as salt-tolerant for cultivation [53]. Selection for salt tolerance in faba bean has been limited because of the lack of reliable and precise measures for salt tolerance criteria [53]. It is anticipated that soil salinity problems will exacerbate because of the wrong agricultural practices [54] along with global climate change [1,55]. Consequently, in addition to amending saline soils, efforts should be made to increase plants’ tolerance to salinity [1]. Antioxidant applications were proved to have a positive influence on a plant’s tolerance to abiotic stresses [56,57,58,59,60].

To our knowledge, there is a noted shortage of data about the influence of AsA, Pro, and GSH including its prospective role in ameliorating salinity stress in strategic crops under large-scale field experiments. Therefore, the aim of the present research is to examine the effects of these antioxidants, at field scale, on salinity tolerance of faba bean plants. The study also aims at comparing the effects of using sequential (AsA-Pro-GSH or GSH-Pro-AsA) vs. singular (AsA, Pro, or GSH) antioxidant foliar application.

## 2. Materials and Methods

### 2.1. Plant Materials, Growth Conditions, and Treatments

Two-season field experiments were conducted consecutively in 2016/17 and 2017/18 at the Experimental Farm of the Faculty of Agriculture, Fayoum University, Southeast Fayoum Governorate (29° 17′ N; 30° 53′ E), Egypt. Healthy seeds of *Vicia faba* L. (CV. Sakha-1; a commercial variety, Sakha Agricultural Research Station, Agricultural Research Centre, Egypt) were sown on 15 and 20 October 2016 and 2017, respectively. Seeds were obtained from Agricultural Research Centre, Giza, Egypt. Healthy, uniform seeds of similar size and color were selected. They were washed with distilled water followed by sterilization in 1% (v/v) sodium hypochlorite for about 2 min. Seeds were then washed thoroughly again with distilled water and left overnight to air-dry at room temperature.

At the recommended planting density of 120 kg ha^−1^, faba bean seeds were sown in 3.0 m × 3.5 m plots, in hills spaced 20–25 cm apart and the rows were spaced 70 cm apart, with a total of 240 plant plot^−1^. Thinning was done before the first irrigation to leave only two plants per hill. According to the recommendations of the Egyptian Ministry of Agriculture and Land Reclamation, soil was supplemented with a complete dose of NPK fertilizer throughout soil preparation and plant growth period. Throughout seed-bed preparation, plants were fertilized with 450 kg ha^−1^ of calcium superphosphate (15.5% P_2_O_5_), 250 kg ha^−1^ of ammonium sulfate (20.5% N), and 120 kg ha^−1^ of potassium sulfate (48% K_2_O). All 100% of the reference crop evapotranspiration (ETo) values from the Fayoum Meteorology Station were used for the calculations of the added irrigation water. In each growing season, plants received seven irrigations through a furrow irrigation system with total water rates of approximately 2800 m^3^ ha^−1^. Agricultural practices were as recommended by the Egyptian Ministry of Agriculture and Land Reclamation. 

The experimental site was the same for both seasons. The methods of [61,62] were carried out for soil analyses and the analysis data are presented in Table 1. Based on its EC values, the soil is classified as strongly saline according to [63]. The treatments of the experiments were arranged in a randomized complete block design. At the 20^th^ day, around 8–9 a.m., after sowing (DAS), seedlings in each plot were sprayed to the point of run-off with singular or sequential antioxidant treatments, i.e., tap water as a control, 0.5 mM AsA, 0.5 mM Pro, and 0.5 mM GSH with three replicate plots^−1^. Each plot had six treatments (control, AsA, Pro, GSH, AsA-Pro-GSH, and GSH-Pro-AsA). Sprays were repeated at 30 and 40 DAS with 10-day intervals.

### 2.2. Measurements of Plant Growth, Yield, and Yield Attributes

Fifty days after sowing (DAS)-9 plants were randomly selected from each experimental plot followed by removal of all adhering soil particles by gently shaking the plants before the length of their shoots was measured. Numbers of leaves and branch number plant^−1^ were then counted. The fresh weight of the shoots was recorded, and then they were placed in an oven at 80 °C for 24 h to determine their dry weights afterwards. Leaf areas were measured using leaf area—leaf weight relationship as demonstrated by [64]. Green pods of 10 plants from each experimental plot were harvested repeatedly and number of pods per plant were counted and weighed to calculate the total number of green pods and pods weight per plant and per hectare. At harvest time (15 April 2017 and 20 April 2018), all dry pods were collected and counted before seeds were extracted for weight measurement including 100-seed weight and seed yield per plant. The seed yield per hectare was calculated.

### 2.3. Measurements of Photosynthetic Efficiency and Stomatal Conductance

Measurements of chlorophyll content were conducted with the use of a chlorophyll meter (SPAD-502, Minolta, Japan) on the top third and fourth leaves of 3 plants/experimental plot/ treatment (*n* = 9). Chlorophyll fluorescence was measured in the first fully expanded dark adapted (for at least 20 min) leaf in two sunny days using a portable fluorometer (Handy PEA, Hansatech Instruments Ltd, Kings Lynn, UK). The maximum quantum yield of PS II Fv/Fm was calculated as =(Fm − Fo)/Fm [65]. Performance index of photosynthesis based on the equal absorption (PI _ABS_) was calculated as reported by [66]. Stomatal conductance (gs; mmol m^−2^ S^−1^) was measured thrice at 8:00 h, 10:00 h, and noon using a leaf porometer (Decagon Devices Inc., Pullman, WA, USA).

### 2.4. Membrane Stability Index and Relative Water Content Measurements

Membrane stability indices (MSI) were estimated from 9 samples for each treatment according to [67]. Leaf relative water content (RWC) was measured according to [68]. After excluding the midrib of leaves, fresh mass (FM), turgid mass (TM), and dry mass (DM) of 2 cm leaf discs were recorded. Leaf RWC was calculated according to the following formula:

Leaf RWC (%) = [(FM − DM) ÷ (TM − DM)] × 100 [68].

For the MSI measurement, 0.2 g leaf sample was placed in a test-tube containing 10 mL of distilled water. Tubes were then heated at 40 °C in a water bath for half an hour, and the electrical conductivity (C1) of the solution was recorded using a conductivity bridge. A second sample was boiled at 100 °C for 10 min, and the conductivity was measured (C2). The MSI was calculated using the formula:

MSI (%) = [1 − (C1/C2)] × 100.

### 2.5. Enzymatic Antioxidants Assays

Fresh leaf tissue (0.5 g fresh sample) was used for SOD, CAT, GR and APX extraction, samples were homogenized in 0.1 M ice cold phosphate buffer (pH = 7.5) containing 0.5 mM EDTA with pre-chilled pestle and mortar. Each homogenate was transferred to centrifuge tubes and was centrifuged at 4 °C in a Beckman refrigerated centrifuge for 15 min at 15,000 ×*g*; supernatant was used for enzyme activity assay [23]. The concentration of the extracted protein was determined using the technique reported by [69]. The enzymes activities determinations were performed, and all expressed as μmol min^−1^ mg^−1^ protein. The activity of superoxide dismutase (SOD; EC 1.15.1.1) was assessed from recording an inhibition of cytochrome reduction in nitroblue tetrazolium (NBT) at 540 nm [70]. About 3 mL of reaction mixture, containing 0.1 ml of 200 mM methionine, 0.1 mL of 2.25 mM nitro-blue tetrazolium (NBT), 0.1 mL of 3 mM EDTA, 1.5 mL of 100 mM potassium phosphate buffer, 1 ml distilled water and 0.05 mL of enzyme extraction, were taken in test tubes in duplicate from each enzyme sample. Two tubes without enzyme extract were taken as control. The reaction was started by adding 0.1 mL riboflavin (60 µM) and placing the tubes below a light source of two florescent lamps (15 W) for 15 min. the reaction was stopped by switching off the light and covering the tubes with black cloth. Tubes without enzyme extract developed maximal color. A non-irradiated complete reaction mixture which did not develop color served as blank. Absorbance was recorded at 560 nm and one unit of enzyme activity was taken as the quantity of enzyme which reduced the absorbance reading of samples to 50% in comparison with tubes lacking enzymes. Glutathione reductase (GR; EC 1.6.4.1) activity was determined by measuring the oxidation of NADPH at 340 nm [71]. The reaction mixture contained 1 mL of 0.2 M potassium phosphate buffer (pH = 7.5) containing 0.1 mM EDTA, 0.5 mL of 3 mM DTNB in 0.01 M potassium phosphate buffer (pH = 7.5), 0.1 mL of 2 mM NADPH, 0.1 mL enzyme extract and distilled water to make up a final volume of 2.9 mL. The reaction was initiated by adding 0.1 mL of 2 mM GSSG. The increase in absorbance at 412 nm was recorded at 25 °C over a period of 5 min on a spectrophotometer. Ascorbate peroxidase (APX; EC 1.11.1.11) activity was measured according to [71] by monitoring the rate of ascorbate oxidation at 290 nm (E=2.8 mM^−1^ cm^−1^). The reaction mixture contained 25 mM phosphate buffer (pH = 7), 0.1 mM EDTA, 1 mM H_2_O_2_, 0.25 mM AsA and the enzyme sample. No change in absorption was found in the absence of AsA in the test medium. Catalase (CAT; EC 1.11.1.6) activity was performed by measuring the decomposition rate of H_2_O_2_ at 240 nm [72]. The 3 mL reaction mixture contained 1.5 mL of 100 mM potassium phosphate buffer (pH = 7), 0.5 mL of 75 mM H_2_O_2_, 0.05 mL enzyme extraction and distilled water to make up the volume to 3 mL. The reaction started by adding H_2_O_2_ and with a decrease in absorbance recorded at 240 nm for 1 min. Enzyme activity was computed by calculating the amount of decomposed H_2_O_2_.

### 2.6. Non-Enzymatic Antioxidants

Ascorbate (AsA) was determined using the method of [73], by extracting fresh leaves (1.0 g) in 5% (w/v) trichloroacetic acid (centrifuged at 15,600 ×*g*) at 4°C for 5 min. AsA content was then assayed by the method described by [23]. According to [74], the rapid colorimetric method was used for proline content measurement (in mg 100 g^−1^ DW of leaf) described in [23]. Dried leaf tissue (0.5 g) was homogenized in 10 mL of 3% (*v/v*) sulfosalicylic acid and centrifuged at 10,000 ×*g* for 10 min. The supernatant was mixed with acid-ninhydrin solution, incubated in a water bath at 90 °C for 30 min, cooled in an ice bath, then 5 mL of toluene was added. The toluene phase was then collected carefully into a test tube and read spectrophotometrically at 520 nm.

To determine GSH contents, the methodology of [75] was followed in fresh fully expanded leaves, homogenized in 2% (*v/v*) metaphsphoric acid, then centrifuged at 17,000 ×*g* for 10 min. The supernatant was neutralized by mixing it with 10% (*w/v*) sodium citrate. The assay was conducted in triplicates as described in [23], and absorbance was recorded at 412 nm. Phenolic compounds, i.e., phenolic aglycone, and phenolic glycosides, were extracted from dried tissues according to method adopted by [76], and The Folin–Ciocalteau phenol method [77] was used for phenolic determination.

### 2.7. Statistical Analysis

The experimental layout was randomized complete block design with three replications for each treatment. All data were subjected to an analysis of variance (ANOVA), and differences between the means were compared by Duncan’s multiple range test (*p* ≤ 0.05) using Genstat statistical package (version 11; VSN International Ltd, Oxford, UK).

## 3. Results

### 3.1. Effect of Antioxidants on Growth Characteristics

All antioxidant treatments significantly increased shoot length, shoot FW, shoot DW, and leaves area, as compared to the control (Table 2). Amongst the studied antioxidant treatments, AsA-Pro-GSH treatment gave the highest increases in the tested growth traits (except for the shoot length), including increased shoot length by 12–21%, number of leaves by 31–34%, number of branches by 84–89%, shoot FW by 44–52%, shoot DW by 67–71%, and leaves area by 58–88% compared to the control in the two seasons. GSH treatment was second in order after AsA-Pro-GSH treatment in terms of increased growth parameters (Table 2).

### 3.2. Effect of Antioxidants on Physiological Attributes

Except for the effect of AsA on F_v_/F_m_ and F_v_/F_0_, foliar application with antioxidants, whether singular or in sequence, significantly improved all tested photosynthetic parameters (relative chlorophyll content, F_v_/F_m_, F_v_/F_0_, PI, and stomatal conductance) compared to the control (Table 3). Best results were found with AsA-Pro-GSH, followed by GSH treatment. Sequenced AsA-Pro-GSH treatment significantly increased relative chlorophyll content by 53–65%, F_v_/F_m_ by 2–4%_,_ F_v_/F_0_ by 14–57%_,_ PI by 69–78%, and g_s_ by 39–44%, in both seasons compared to the control (Table 3), whereas GSH significantly increased relative chlorophyll content by 48–50%, F_v_/F_m_ by 2–2.6%, F_v_/F_0_ by 14–52%, PI by 56–80%, and g_s_ by 37–41% compared to the control (Table 3). Pro treatment resulted in the lowest stomatal conductance among antioxidant treatments (Table 3). All antioxidant treatments significantly increased leaf RWC% and WUE compared to the control (Table 4). The best improvements were found in plants treated with sequenced AsA-Pro-GSH as it increased leaf RWC% by 19–22%, MSI by 19–23%, and WUE by 65–70%, compared to the control in both seasons (Table 4).

### 3.3. Enzymatic and Non-Enzymatic Antioxidants

**Figure 1:**
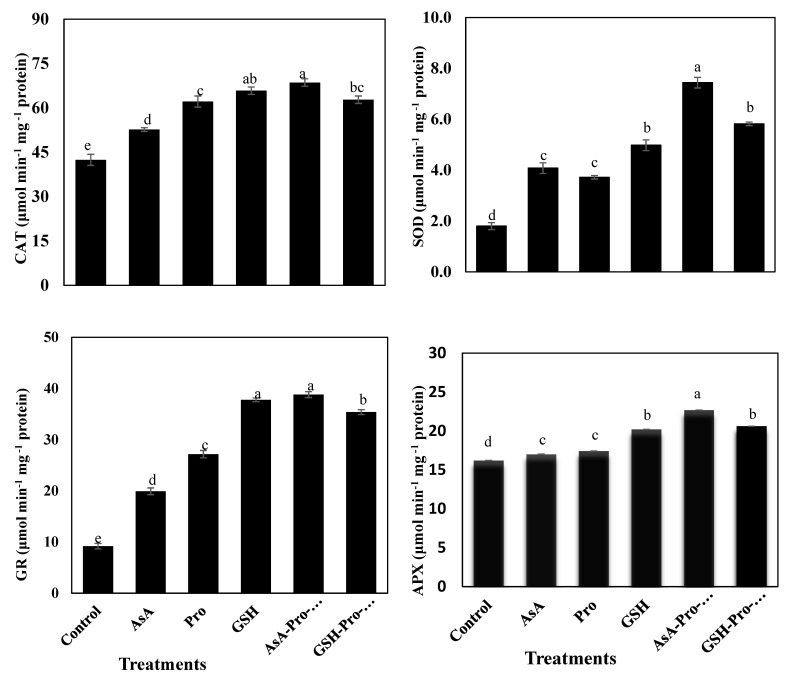
Effect of foliar application of singular and sequential antioxidants on enzymatic antioxidants; catalase (CAT), superoxide dismutase (SOD), glutathione reductase (GR) and ascorbate peroxidase (APX) antioxidants of *Vicia faba* L. plants grown in saline soil conditions. Vertical bars represent means of 5 replications ± S.E (*p* ≤ 0.05). Columns marked by different letters are significantly different.

**Figure 2:**
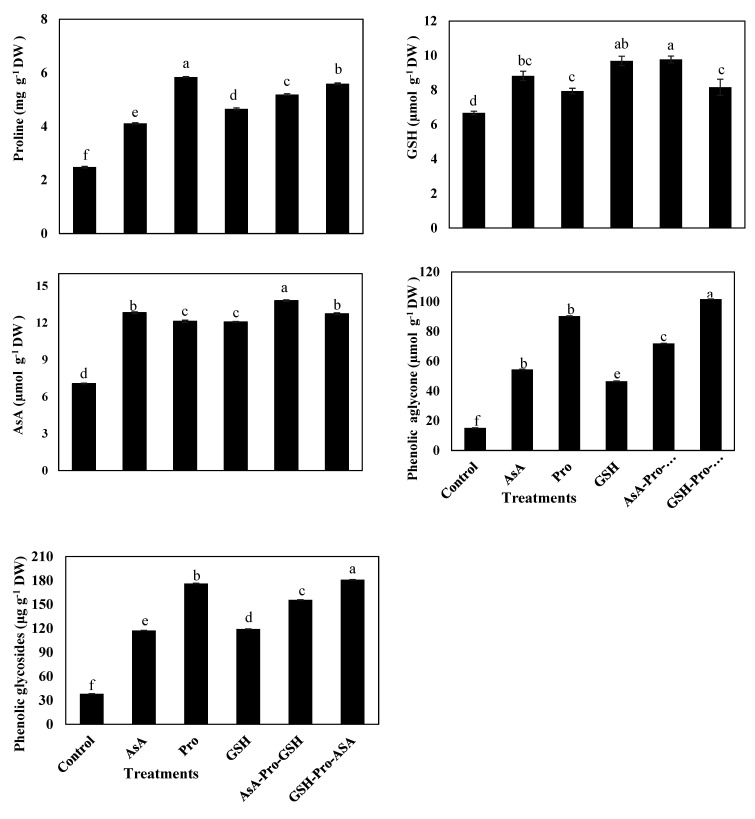
Effect of foliar application of singular and sequential antioxidants on non-enzymatic antioxidants; free proline, glutathione (GSH), ascorbic acid (AsA), aglycones antioxidants and phenolic glycosides of *Vicia faba* L. plants grown in saline soil conditions. Vertical bars represent means of 5 replications ± S.E (*p* ≤ 0.05). Columns marked by different letters are significantly different.

Enzymatic activity levels of GR, CAT, SOD, and APX were significantly increased by all antioxidant treatments compared to the control (Figure 1). The highest significant levels of GR, CAT, SOD, and APX activities were found in plants treated with sequenced AsA-Pro-GSH. GSH treatment came in second place after AsA-Pro-GSH for enhancing the activity levels of GR, CAT, and APX (Figure 1). Contents of Pro, AsA, GSH, phenolic aglycone, and phenolic glycosides were significantly increased by all antioxidant treatments compared to the control (Figure 2). The highest content of AsA was obtained from AsA-Pro-GSH treatment, while that of GSH was obtained from both AsA-Pro-GSH and GSH treatments. GSH-Pro-AsA treatment provided the highest contents of phenolic aglycone and phenolic glycosides. Pro content was significantly enhanced with Pro treatment, followed by GSH-Pro-AsA (Figure 2).

### 3.4. Effect of Antioxidants on Yield and Yield Attributes

All antioxidant treatments significantly increased green pods weight/plant, green pods yield/hectare, and seed yield/hectare in the two seasons (Table 5 and Table 6). The best results were obtained with AsA-Pro-GSH treatment as it significantly increased the number of green pods by 48–56%, green pods weight/plant by 31%, green pods yield/hectare by 35–39%, biological yield/hectare by 42–46%, straw yield/hectare by 29–39%, seed yield/hectare by 67–70%, average 100-seed weight by 5.5–6.8%, and HI% by 10–19%, compared to the control in both seasons (Table 5 and Table 6). The second-best results were achieved by the GSH treatment after AsA-Pro-GSH as the corresponding values significantly increased by 44–52%, 29%, 34%, 27–32%, 14–22%, 54–57%, 4–5%, and 21–24%, respectively (Table 5 and Table 6).

## 4. Discussion

### 4.1. Improvement of Salinity Tolerance by Integrating Three Powerful Antioxidants into One Sequential Treatment

In the current experiment, antioxidant treatments (singular or sequenced) were applied to faba bean plants to examine their effects on plant tolerance to salinity stress. The results of the present study showed that most antioxidant treatments significantly improved most of the studied plant characteristics compared to the control treatment (Table 2, Table 3, Table 4, Table 5 and Table 6). The sequenced AsA-Pro-GSH treatment, followed by the singular GSH treatment gave significantly and consistently better results than the control treatment and most of the other antioxidant treatments. 

The observed benefit of starting a sequenced antioxidant treatment with AsA can be explained as AsA is one of the essential antioxidant enzymes that detoxify ROS against oxidative stress. Apoplastic AsA can function in signaling for adaptation responses against environmental stresses. The antioxidant defenses at the apoplast are alarmed upon the identification of an elicitor molecule by the proper receptor molecule [78]. Plants with high AsA contents are able to tolerate different stress conditions [79], while those with poor intrinsic AsA are sensitive to stressful conditions [80]. Pro, the second antioxidant in the sequenced AsA-Pro-GSH treatment, is an amino acid that occurs in higher plants, and accumulates in larger amounts for adaptive responses to abiotic stresses [41,81]. Accumulation of Pro is strongly correlated with stress tolerance and adaptation to metabolic disturbance [82]. Internal levels of Pro in plants depend on its biosynthesis, degradation, and transport between cells and between cellular compartments [83]. It helps plants recover from stress rapidly, and protects plants from stress by acting as a scavenger for ROS and/or enzyme protectant [83]. Upon relief from stress, Pro breaks down and may provide an important energy for mitochondrial phosphorylation and ATP generation to help repair the oxidative damage [84]. Pro is one of the compatible solutes that is normally accumulated in the cytosol and organelles acting as an osmolyte for osmotic adjustment under osmotic stresses [11]. Upon salt stress, the expression of salt-responsive genes is up regulated for induction of Pro biosynthesis, accumulation, and increase its concentrations in the leaves, stems, and roots [81].

GSH, whether as a third component of AsA-Pro-GSH sequenced treatment or as a singular treatment, has helped in improving plants’ tolerance. It is another antioxidant that functions as a free radical scavenger for superoxide radical, hydroxyl radical, and singlet oxygen [85]. It is particularly important because of the non-protein thiol group which makes it suitable for many signaling pathways and biochemical functions [86]. It can contribute to the regeneration of ascorbate through the AsA–GSH cycle which gives GSH an extra powerful role in the antioxidative defense [86]. It has been demonstrated that the AsA–GSH cycle improved the tolerance of lentils to salinity as salinized plants had higher ascorbate peroxidase and glutathione reductase enzyme activities to scavenge the ROS generated from the salt stress shock [87].

### 4.2. Sequential AsA-Pro-GSH Improves Photosynthetic Efficiency and Relative Chlorophyll Content

Sequential AsA-Pro-GSH or proline, and GSH singular treatment, significantly improved photosynthetic efficiency (*F_v_/F_m_*, *F_v_/F_0_*, and PI) and relative chlorophyll content when compared with the control. However, this improvement could not be noted with AsA treatment on *F_v_/F_m_* and *F_v_/F_0_* (Table 3). In accordance with our results, [80] found that Arabidopsis plants with impaired AsA–GSH cycle and low intrinsic AsA had a decreased photosynthetic efficiency and chlorophyll contents [80], emphasizing the importance of AsA in maintaining the photosynthetic efficiency. Chloroplasts, the major sites of photosynthesis, are very sensitive to environmental stresses and consequently are the main sites for ROS generation [20,88,89]. AsA is found in high concentrations in chloroplasts and is believed to have key functions in photosynthesis protection from oxidative damage [90]. Several roles of ascorbate in photosynthesis have been proposed. AsA is essential for the Mehler peroxidase reaction that is known to be a powerful detoxification system in chloroplasts [91]. It is also essential for scavenging the generated harmful ROS byproducts of photosynthesis [92].

Integrating Pro in a sequenced AsA-Pro-GSH is helpful for improving photosynthetic efficiency and relative chlorophyll content (SPAD value). It was reported that Pro mitigates the effects of salinity on photosynthesis by efficiently maintaining the mitochondrial electron transport complex II and activity of enzymes such as RUBISCO [83]. Likewise, salt-stressed melon plants treated with Pro had higher photosynthetic efficiency and chlorophyll content [56]. GSH is naturally available in high concentrations in the chloroplast in its reduced form and it has a key function in the protection from oxidative damage [20,86]. Therefore, whether used alone or in a sequenced treatment, GSH could alleviate the negative effects of salinity stress on photosynthetic efficiency. In tomato plants, GSH treatment was able to alleviate salinity stress effects by increasing *F_v_/F_m_*, the photochemical activity, and the photosynthetic electron transport rate of PSII by dissipating heat, to protect the photosynthetic apparatus from the excess excitation energy and the risk of ROS generation [88].

### 4.3. Application of AsA-Pro-GSH Increases Enzymatic Antioxidant Levels for Better Defense System

In the present experiment, SOD and APX levels were significantly the highest with the application of the sequenced AsA-Pro-GSH treatment, whereas the highest levels of CAT and GR level were achieved by both AsA-Pro-GSH sequence treatment and GSH singular treatment (Figure 1). In normal conditions, chloroplast use sun light energy to oxidize H_2_O while oxygen is a byproduct of photosynthesis that takes place at PSII. The electrons are directed to NADP^+^, which is reduced to NADPH, to be used in CO_2_ assimilation. Part of the electron flow is used by the Mehler reaction for the reduction of O_2_ by PSI to superoxide (O_2_^•^) [91]. GR assures the availability of NADP^+^ as an electron donor to minimize the formation of O_2_^•^. Additionally, GR keeps a high GSH:GSSG ratio that is required for AsA generation [86].

The existence of the two regeneration systems (Mehler reactions and the AsA-GSH cycle) assure the effective regeneration of the reduced AsA [25,80]. The Mehler reaction is limited to the chloroplast, whereas the AsA–GSH cycle is located in many cell compartments [25]. Unlike other enzymes, CAT is a major antioxidant that does not need a reductant for the H_2_O_2_ dismutation reaction [86,93].

In the present experiment, plants treated with AsA-Pro-GSH and GSH treatments had significantly the highest concentrations of non-enzymatic antioxidants such as GSH. AsA concentration in the plants was the highest with AsA-Pro-GSH treatment (Figure 2). These results are in agreement with those reported by [23] on cucumber plants grown under cadmium stress conditions. In salinized maize plants, AsA concentrations were the highest with AsA-Pro-GSH treatment, whereas GSH concentration was the highest with both GSH-Pro-AsA and AsA-Pro-GSH treatments [94]. In our experiment, Pro concentration was the highest with Pro treatment (Figure 2). This is contrary to results by [94], where salinized maize plants had the highest Pro concentration with AsA-Pro-GSH treatment. 

### 4.4. Application of AsA-Pro-GSH Alleviates Membrane Damage and Enhances Water Status of Plants

Sequenced AsA-Pro-GSH treatment gave significantly higher leaf MSI%, RWC%, and WUE values than the control and gave the highest values among antioxidant treatments (Table 4). When plants are salt-stressed, AsA may help alleviate the membrane damage caused by ROS scavenging. It could inhibit lipid peroxidation or bind to membrane lipids to stabilize plasma membranes. Consequently, AsA could maintain membrane permeability, functions, and properties under salt stress conditions [95]. Additionally, AsA can develop tocopherol from tocopheroxyl radicals, adding to the membrane protection [86]. In our experiment, the observed improved MSI% and water status of plants is indicative of the enhanced membrane integrity by AsA application. 

In salinized Vicia faba plants, Pro application was found to enhance membrane stability, reduce water efflux, improve leaf RWC% [83,96]. GSH application was found to maintain membrane integrity and cell viability of salinized onion plants through scavenging of ROS and hence prevention of lipid peroxidation [97]. In salinized lentil plants, GSH was also associated with more RWC% [87].

### 4.5. Application of AsA-Pro-GSH Enhances Stomatal Conductance

Reactive oxygen species are also involved in the regulation of stomatal aperture [98]. Under stressful environmental conditions, H_2_O_2_ is generated and works as an intermediate in ABA signaling of the guard cells of plants [99]. Hydrogen peroxide was found to induce closure of stomata of *Vicia faba* plants which was reversed by the exogenous application of AsA because of its crucial role in detoxifying generated H_2_O_2_ [99]. The authors in [100] reported that GSH treatment had a direct effect on ABA biosynthesis and signaling, and the upregulation of ABA-responsive genes such as OPEN STOMATA1 (OST1/ SnRK2.6) [100]. Exogenous GSH was found to overcome stomatal limitations, increase photochemical quenching, and improve light use efficiency of tomato plants [88]. 

Singular Pro treatment, however, resulted in the lowest stomatal conductance increases amongst the studied antioxidant treatments in the present study. Pro levels in the leaves were correlated with stomatal resistance of salt-stressed *Commelina communis* plants [101], and a strong relationship was found between exogenous Pro application and stomatal resistance of *Vicia faba* plants [102]. In the sequenced AsA-Pro-GSH treatment of the current experiment, however, it seems that the small increases in stomatal conductance by Pro were neutralized with the stimulating effects of AsA and GSH which resulted in the highest stomatal conductance among the studied treatments (Table 3). 

### 4.6. Integration of AsA-Pro-GSH Promotes the Overall Growth and Yield

Most of the antioxidant treatments gave significantly better growth and yield results than the corresponding control (Table 2, Table 5 and Table 6). The positive effects were more pronounced when starting with AsA along with the integration of Pro and GSH in a sequenced AsA-Pro-GSH treatment than most of the other treatments. Similarly, [23], reported that application of AsA-Pro-GSH under cadmium stress conditions gave significantly higher growth traits than the control. 

Research studies on transgenic and/or mutant plants showed that AsA is essential for the growth, development, and tolerance to salinity [79,80,103]. Transgenic plants overexpressing AsA peroxidase had better growth and development than the mutant or the wild-type plants under salt stress conditions [104]. AsA contributes to promoting plant growth by enhancing cell division at the meristems and cell elongation at the differentiated tissues [24]. AsA is involved in the modulation of cell growth as it regulates the biosynthesis of hydroxyproline-rich gylcoproteins, which is essential for the development of G1 and G2 cell cycle phases. AsA is also involved in the redox reaction at the plasma membrane required for cell elongation [105]. The changes from AsA to DHA also seem to be vital in the process of cell elongation [106]. 

Exogenous application of Pro to stressed plants has been found to enhance plant growth and other physiological parameters [47,83]. Similar to our results, Pro was found to improve salt tolerance by significantly improving free proline concentrations, growth, yield, and yield components of lupines plants [39]. In salt-stressed canola plants, exogenous application of Pro increased their tolerance via the enhancement in stem length, leaves area, shoot dry weight, components of the yield and consequently seed yield [107]. Pro also alleviated the growth inhibition of salt-stressed cucumber plants, which was associated with higher RWC% and decreased SOD activity [108]. 

Exogenous application of GSH to salinized wheat plants enhanced seedling growth, plant height, fresh and dry weights [58]. In the current research, singular GSH treatment was mostly second after the sequenced AsA-Pro-GSH in terms of alleviating salt stress effects and in improving the growth traits (Table 2), physiological parameters (Table 3 and Table 4), and yield and yield components (Table 5 and Table 6). A previous study has reported that exogenous application of GSH to salt-stressed plants improved salinity tolerance [59]. The authors in [59] attributed that improvement to the reduced production and accumulation of ROS which resulted in better antioxidant hemostasis and reduced oxidative damage, with a consequent improvement in photosynthesis, growth and yield compared to control plants.

## 5. Conclusions

Foliar application of singular or sequential AsA, Pro, and GSH has positive effects on the tolerance of faba bean plants to salinity in terms of enhancing growth characteristics, chlorophyll content, photosynthetic efficiency, stomatal conductance (gs), plant water status, and yield and its components along with the improvement of enzymatic and non-enzymatic antioxidant levels. That improvement was most remarkable with the use of sequenced AsA-Pro-GSH, followed by the singular GSH treatment. AsA-Pro-GSH may offer a potential economic alternative for salinity-stress alleviation in faba bean plants. However, further studies are needed to show the benefits of using sequential antioxidant treatments on other strategic crops in large-scale field experiments.

## Figures and Tables

**Table 1:** Physical and chemical properties of the studied soils.

Layer (cm)	Particle Size Distribution				Bulk density g cm^−3^	K_sat_ cm h^−1^	Soil Moisture Content AT			pH	ECe dS m^−1^	CaCO_3_, %	OM %
	Sand %	Silt %	Clay %	Texture Class			F.C %	W.P %	A.W %				
0–20	65.07	16.08	18.85	S.L.	1.44	2.21	23.00	10.02	12.98	7.63	4.3	7.60	0.96
20–40	71.62	12.09	16.29	S.L.	1.47	2.01	20.72	9.15	11.57	7.60	4.5	6.4	0.83
40–60	73.61	12.15	14.24	S.L.	1.56	1.89	18.71	8.05	10.66	7.43	4.8	6.20	0.51

S.L. = sandy loam, F.C = field capacity, W.P = wilting point, A.W= available water and K_sat_ = hydraulic conductivity and OM = organic matter.

**Table 2:** Effect of the foliar application of singular and sequential antioxidants on growth characteristics of *Vicia faba* L. plants grown in saline soil conditions during 2016/17 (SI) and 2017/18 (SII) seasons.

Treatments	Shoot Length (cm)	No. of Leaves Plant^−1^	No. of branches Plant^−1^	Shoot FW (g)	Shoot DW (g)	Leaves area (dm^2^)
**SI**						
Control	87.0 ± 2.9 b	50.5 ± 1.0 c	2.25± 0.25 b	161.9 ± 3.7 c	19.5 ± 0.9 c	28.9 ± 1.40 d
AsA	108.0 ± 2.5 a	57.0 ± 2.7 bc	3.50± 0.65 ab	210.6 ± 2.1 b	27.1 ± 0.3 b	42.5 ± 0.84 c
Pro	105.2 ± 2. 8 a	58.3 ± 1.2 bc	3.75± 0.25 a	216.2 ± 1.6 b	26.0 ± 1.4 b	43.5 ± 1.60 bc
GSH	111.2 ± 3.6 a	64.8 ± 0.9 ab	4.00± 0.41 a	230.2 ± 2.0 ab	30.3 ± 1.1 ab	48.6 ± 1.43 b
AsA-Pro-GSH	105.0 ± 3.8 a	67.5 ± 1.0 a	4.25± 0.48 a	245.7 ± 2.2 a	33.3 ± 0.2 a	54.4 ± 0.67 a
GSH-Pro-ASA	102.5 ± 1.2 a	62.3 ± 1.7 ab	3.50± 0.29 ab	222.5 ± 2.4 ab	28.1 ± 0.8 b	45.3 ± 2.48 bc
**SII**						
Control	94.5 ± 1.9 b	50.0 ± 1.3 d	2.50±0.29 b	168.9 ± 2.5 d	20.6 ± 1.8 c	32.7 ± 2.18 d
AsA	111.0 ± 3.4 a	58.1 ± 1.2 c	3.86±0.43 a	210.7 ± 2.4 c	26.9 ± 0.45 ab	42.2 ± 2.01 bc
Pro	102.5 ± 3.3 ab	58.8 ± 1.5 bc	3.80±0.27 a	214.1 ± 3.7 bc	27.0 ± 0.90 a	42.4 ± 0.26 c
GSH	110.2 ± 4.8 a	62.5 ± 0.9 a	4.00±0.01 a	228.1 ± 3.6 ab	29.4 ± 0.99 a	49.0 ± 2.23 ab
AsA-Pro-GSH	106.2 ± 2.4 a	65.3 ± 1.0 a	4.6±0.12 a	243.4 ± 4.3 a	34.3 ± 0.68 a	51.8 ± 2.52 a
GSH-Pro-ASA	101.0 ± 2.5 ab	59.5 ± 0.5 a	3.83±0.28 a	225.7 ± 9.5 bc	27.8.0 ± 1.8 a	45.1 ± 2.02 bc

Differences between mean values (*n* = 9 ± SE) followed by the same letter in each column are not significant by Duncan’s multiple range test at *p* ≤ 0.05.

**Table 3:** Effect of singular and sequential antioxidants foliar application on relative chlorophyll content (SPAD value), photosynthetic efficiency (*F_v_/F_m_*, *F_v_/F_0_*, and PI) and stomatal conductance (g_s_) of *Vicia faba* L. plants grown in saline soil conditions during 2016/17 (SI) and 2017/18 (SII) seasons.

Treatments	SPAD Value	*F_v_/F_m_*	*F_v_/F_0_*	PI	gs (mmol m^−2^ S^−1^)
**SI**					
Control	31.98 ± 4.2 c	0.822 ± 0.007 b	4.60 ± 0.16 b	3.04 ± 0.27 c	122.9 ± 2.1 c
AsA	40.46 ± 1.4 b	0.827 ± 0.004 ab	4.82 ± 0.12 ab	4.59 ± 0.15 b	172.0 ± 2.5 a
Pro	40.14 ± 2.2 b	0.836 ± 0.004 a	5.13 ± 0.13 a	4.81 ± 0.19 ab	152.1 ± 1.9 b
GSH	47.88 ± 1.4 a	0.839 ± 0.004 a	5.25 ± 0.13 a	5.46 ± 0.19 a	167.8 ± 1.7 a
AsA-Pro-GSH	48.78 ± 0.5 a	0.840 ± 0.004 a	5.23 ± 0.16 a	5.40 ± 0.27 a	171.4 ± 1.2 a
GSH-Pro-ASA	43.38 ± 1.5 ab	0.839 ± 0.005 a	5.24 ± 0.18 a	4.88 ±0.31 ab	163.1 ± 1.8 a
**SII**					
Control	29.90 ± 1.6 c	0.807 ± 0.004 b	3.34 ± 0.43 b	3.14 ± 0.48 c	131.9 ± 2.2 d
AsA	43.46 ± 2.0 b	0.825 ± 0.004 a	4.89 ± 0.16 ab	4.15 ± 0.28 b	174.5 ± 2.8 bc
Pro	44.80 ± 1.6 ab	0.834 ± 0.007 a	4.42 ± 0.12 a	4.56 ± 0.39 ab	163.9 ± 0.3 c
GSH	44.32 ± 1.6 ab	0.828 ± 0.007 a	5.07 ± 0.21 a	4.90 ± 0.66 a	186.0 ± 2.6 ab
AsA-Pro-GSH	49.24 ± 0.87 a	0.840 ± 0.002 a	5.24 ± 0.18 a	5.31 ± 0.34 a	190.3 ± 0.8 a
GSH-Pro-ASA	42.94 ± 2.6 b	0.825 ± 0.006 a	4.76 ±0.20 a	4.65 ± 0.42 ab	178.0 ± 1.6 ab

Differences between mean values (*n* = 9 ± SE) followed by the same letter in each column are not significant by Duncan’s multiple range test at *p* ≤ 0.05.

**Table 4:** Effect of singular and sequential antioxidants foliar application on plant water status (relative water content (RWC %) and membrane stability index (MSI %)), and water use efficiency (WUE) of *Vicia faba* L. plants grown in saline soil conditions during 2016/17 (SI) and 2017/18 (SII) seasons.

Treatments	RWC %	MSI %	WUE (Kg m^3^)
**SI**			
Control	77.0 ± 0.57 c	64.9 ± 0.82 c	0.56±0.01 d
AsA	85.1 ± 0.76 b	69.7 ± 2.5 bc	0.75±0.00 c
Pro	87.4 ± 1.5 ab	69.3 ± 2.6 bc	0.75±0.02 c
GSH	86.8 ± 1.5 b	67.1 ± 1.1 bc	0.87±0.02 b
AsA-Pro-GSH	91.9 ± 0.57 a	77.3 ± 1.6 a	0.95±0.01 a
GSH-Pro-ASA	88.9 ± 1.8 ab	72.7 ± 3.7 ab	0.78±0.01 c
**SII**			
Control	75.3 ± 0.77 c	63.1 ± 3.1 c	0.55±0.02 d
AsA	83.9 ± 2.1 b	72.0 ± 1.7 ab	0.78±0.02 bc
Pro	84.2 ± 2.6 b	68.0 ± 2.6 bc	0.75±0.00 c
GSH	87.1 ± 2.7 ab	73.9 ± 0.94 ab	0.85±0.03 ab
AsA-Pro-GSH	92.0 ± 1.8 a	77.7 ± 1.8 a	0.91±0.02 a
GSH-Pro-ASA	86.2 ±1.4 ab	71.0 ±1.5 ab	0.81±0.03 bc

Differences between mean values (*n* = 9 ± SE) followed by the same letter in each column are not significant by Duncan’s multiple range test at *p* ≤ 0.05.

**Table 5:** Effect of foliar application of singular and sequential antioxidants on green pods yield of *Vicia faba* L. plants grown in saline soil conditions during 2016/17 (SI) and 2017/18 (SII) seasons.

Treatments	No. of Pods Plant^−1^	Pods Weight Plant^−1^ (g)	Pods Yield Hectare^−1^ (ton)
**SI**			
Control	10.8 ± 0.48 d	109.5 ± 1.8 c	8.8 ± 1.03 c
AsA	13.3 ± 0.85 bc	125.5 ± 4.6 b	10.5 ± 0.19 b
Pro	12.3 ± 0.25 cd	123.0 ± 4.2 b	10.6 ± 0.25 b
GSH	15.5 ± 1.79 ab	141.7 ± 2.6 a	11.8 ± 0.63 a
AsA-Pro-GSH	16.8 ± 1.08 a	143.0 ± 2.9 a	12.2 ± 0.83 a
GSH-Pro-ASA	13.8 ±1.38 bc	135.1 ± 4.69 ab	11.2 ± 0.89 ab
**SII**			
Control	10.50 ± 1.2 b	109.5 ± 1.8 c	8.9 ± 0.15 c
AsA	12.75 ± 1.1 ab	125.5 ± 4.6 b	10.5 ± 0.38 b
Pro	13.25 ± 1. 8 ab	123.0 ± 4.2 b	10.3 ± 0.35 b
GSH	16.00 ± 0.7 a	141.7 ± 2.6 a	11.9 ± 0.22 a
AsA-Pro-GSH	15.50 ± 1.2 a	143.0 ± 2.9 a	12.0 ± 0.24 a
GSH-Pro-ASA	13.75 ± 0.9 ab	135.1 ± 4.6 ab	11.4 ± 0.39 a

Differences between mean values (*n* = 10 ± SE) followed by the same letter in each column are not significant by Duncan’s multiple range test at *p* ≤ 0.05.

**Table 6:** Effect of foliar application of singular and sequential antioxidants on yield and its components of *Vicia faba* L. plants grown in saline soil conditions during 2016/17 (SI) and 2017/18 (SII) seasons.

Treatments	Biological Yield Hectare^−1^ (ton)	Straw Yield Hectare^−1^ (ton)	Seed Yield Hectare^−1^ (ton)	100-Seed Weight Average	HI (%)
**SI**					
Control	7.9 ± 0.45 b	5.6 ± 0.24 b	2.3 ± 0.06 c	90.2 ± 0.21 c	29.4 ± 0.3 e
AsA	9.3 ± 0.43 ab	6.2 ± 0.21 ab	3.1 ± 0.01 b	93.9 ± 0.27 ab	33.2 ± 0.6 cd
Pro	9.7 ± 0.49 ab	6.7 ± 0.23 ab	3.1 ± 0.09 b	92.4 ± 0.07 b	31.7 ± 0.6 d
GSH	10.0 ± 0.77 ab	6.4 ±0.18 b	3.6 ± 0.07 a	95.0 ± 0.51 a	36.4 ±1.1 a
AsA-Pro-GSH	11.2 ± 0.63 a	7.23 ± 0.23 a	3.9 ± 0.06 a	95.2 ± 0.35 a	35.1 ±0.5 ab
GSH-Pro-ASA	9.2 ± 0.36 ab	6.1 ± 0.31 ab	3.2 ± 0.06 b	93.3 ± 0.21 b	34.6 ±0.8 bc
**SII**					
Control	7.6 ± 0.07 d	5.4 ± 0.07 c	2.21± 0.07 c	91.3 ± 0.18 b	29.3 ± 1.8 b
AsA	9.4± 0.03 c	6.3 ± 0.07 bc	3.2 ± 0.07 ab	92.2 ± 0.94 b	33.6 ± 0.8 ab
Pro	9.0 ± 0.13 c	6.0 ± 0.14 bc	3.0 ± 0.01 b	92.4 ± 0.94 b	33.7 ± 0.60 ab
GSH	10.0 ± 0.09 b	6.6 ± 0.05 ab	3.4 ± 0.12 ab	94.6 ± 1.24 ab	35.5 ± 1.6 ab
AsA-Pro-GSH	11.1 ± 0.07 a	7.5 ± 0.13 a	3.7 ± 0.09 a	97.5 ± 0.55 a	32.3 ± 0.65 ab
GSH-Pro-ASA	9.6 ± 0.24 c	6.2 ± 0.03 bc	3.4 ± 0.12 ab	94.2 ± 0.78 ab	36.4 ± 0.34 a

Differences between mean values (*n* = 9 ± SE) followed by the same letter in each column are not significant by Duncan’s multiple range test at *p* ≤ 0.05.

## Data Availability

All the data generated or analyzed during the current study are included in the published article.
